# Large Language Models for Clinical Data Extraction in Workplace Injury Rehabilitation: Protocol for a Retrospective Pilot Study of Accuracy, Fairness, and Methodological Considerations in Workers’ Compensation Medical Chart Review

**DOI:** 10.2196/99807

**Published:** 2026-09-22

**Authors:** Armaan Rehman Shah, Barbara Gorczyca Abel, Majid Komeili, Basem Gohar, Behdin Nowrouzi-Kia

**Affiliations:** 1 Department of Occupational Science and Occupational Therapy Temerty Faculty of Medicine University of Toronto Toronto, ON Canada; 2 Institute for Better Health Trillium Health Partners Mississauga, ON Canada; 3 School of Computer Science Carleton University Ottawa, ON Canada; 4 Department of Population Medicine University of Guelph Guelph, ON Canada; 5 Centre for Research in Occupational Safety and Health Laurentian University Sudbury, ON Canada; 6 Krembil Research Institute-University Health Network Toronto Western Hospital Toronto, ON Canada

**Keywords:** artificial intelligence, AI, large language model, LLM, natural language processing, occupational rehabilitation, workplace injury, workers’ compensation, algorithmic fairness, return to work

## Abstract

**Background:**

Electronic medical charts within Workplace Safety and Insurance Board (WSIB) specialty programs contain rich but unstructured clinical and sociodemographic data essential for injury classification, treatment planning, and compensation decisions. Manual chart review is time-consuming, inconsistent, and susceptible to reviewer bias. Large language models (LLMs) extract complex information from unstructured clinical text, yet their application to workplace injury rehabilitation remains unexplored.

**Objective:**

This study will evaluate the accuracy, fairness, and methodological implications of using LLMs to extract clinical and rehabilitative information from WSIB medical charts at Trillium Health Partners (THP), Canada. We will assess model performance, examine algorithmic bias across demographic subgroups, and explore ethical implications for clinical decision-making and workplace compensation.

**Methods:**

We will conduct a retrospective review of 50 medical charts from the WSIB Back and Neck specialty program at THP, spanning January 2018 to December 2024. General-purpose models (Qwen3-VL-8B and InternVL3.5-8B) and domain-specific biomedical models (MedGemma-27B and LLaMA-3-Meditron-8B) will be evaluated off the shelf using zero-shot and few-shot prompting; the biomedical models will also be fine-tuned. Charts will be partitioned at the chart level into development, training, and held-out test sets, preventing leakage across purposes. Ground truth will be established by independent human annotation of all 50 charts, with interannotator agreement quantified using Cohen κ. The primary outcome is the macroaveraged *F*_1_-score across categorical variables on the held-out test set; secondary outcomes are the per variable *F*_1_-score, mean absolute error and root mean squared error for continuous variables, and span-level *F*_1_-score. Progression to the larger study requires a macroaveraged *F*_1_-score of at least 0.80, a pragmatic feasibility criterion; fairness and continuous-variable results are supporting outcomes. Algorithmic fairness will be examined using demographic parity and equalized odds across subgroups. A secure hybrid architecture will keep all identifiable personal health information within the THP infrastructure, with cloud compute restricted to transient processing.

**Results:**

This study was funded by the Data Sciences Institute at the University of Toronto in April 2025; the award supported protocol development and ended on April 30, 2026. As of August 12, 2026, neither had a research ethics application been submitted nor had any chart been accessed. Applications to the THP and University of Toronto research ethics boards are anticipated in late 2026, and no data will be retrieved or processed before approval from both boards. Contingent on approval and further funding, data collection is anticipated through 2027, analysis in late 2027 to early 2028, and results submitted for publication in 2028.

**Conclusions:**

This protocol describes the first systematic evaluation of LLM-based data extraction applied to workplace injury medical charts. Findings will inform best practices for the responsible, reproducible, and equitable integration of these tools into occupational health and workers’ compensation decision-making.

**International Registered Report Identifier (IRRID):**

PRR1-10.2196/99807

## Introduction

Workplace injuries impose a substantial burden on individuals, employers, and health care systems worldwide. Successful return-to-work (RTW) outcomes are essential for minimizing long-term physical, mental, and economic consequences for injured workers [1,2]. Clinical decision-making in workplace injury rehabilitation depends heavily on accurate assessment of injury severity, functional limitations, treatment pathways, and prognostic factors documented in electronic medical charts, and the effectiveness of rehabilitation and RTW interventions rests on how well this clinical information is captured and applied [3]. Within Ontario, Canada, the Workplace Safety and Insurance Board (WSIB) administers specialty recovery programs that provide targeted rehabilitation for workers with approved workplace injury claims [4].

Electronic health records (EHRs) within WSIB specialty programs contain rich clinical and sociodemographic information, including diagnoses, injury mechanisms, occupational duties, treatment plans, and RTW barriers. However, extracting structured data from these records is a resource-intensive process that traditionally relies on manual chart reviews by trained clinicians or research personnel. Manual review is time-consuming, susceptible to human bias and inconsistency, and poorly scalable to large datasets [5,6]. These limitations constrain researchers’ and decision-makers’ ability to leverage clinical data for quality improvement, outcome evaluation, and equitable service delivery.

Recent advances in artificial intelligence (AI), particularly large language models (LLMs), offer a promising approach to automating the extraction of structured information from unstructured clinical text [7,8]. Transformer-based models, such as Bidirectional Encoder Representations from Transformers for Biomedical Text Mining (BioBERT), Clinical Bidirectional Encoder Representations from Transformers (ClinicalBERT), and Medical Bidirectional Encoder Representations from Transformers (Med-BERT), have demonstrated effectiveness in extracting diagnoses, medications, and symptoms from EHRs [9-11]. Newer general-purpose LLMs, including Qwen3-VL-8B [12] and InternVL3.5-8B [13], have shown improved contextual reasoning and adaptability to previously unseen clinical tasks through zero-shot and few-shot learning, and general-purpose models of this class have performed competitively on medical reasoning benchmarks [14]. More recent domain-specific models, such as MedGemma-27B [15] and LLaMA-3-Meditron-8B [16], the latter continuing a line of medical pretraining that began with MEDITRON [17] on the LLaMA (Large Language Model Meta AI) architecture [18], have been developed with a focus on biomedical and clinical reasoning, enabling improved performance on tasks involving diagnosis extraction, symptom identification, and interpretation of EHRs. Bannett et al [19] demonstrated that LLMs can identify clinically relevant information in pediatric medication monitoring records, with accuracy comparable to clinician review, supporting the potential for hybrid human-AI workflows in chart review contexts.

Despite growing evidence supporting the application of LLMs in clinical data extraction across various medical domains, no existing study has systematically evaluated how LLMs perform in reviewing WSIB-related patient records. Workplace injury medical charts pose unique challenges not addressed in prior research, including documentation of occupational duties and demands, injury mechanisms, RTW barriers, functional capacity evaluations, and multidisciplinary rehabilitation plans. Furthermore, the potential for algorithmic bias in AI-driven extraction from occupational health records, which directly affects worker compensation and access to rehabilitation, has not been critically examined [20,21].

The evidence base for clinical extraction has grown quickly but remains concentrated in a small number of settings. A systematic review of generative LLM applications to EHR data found that evaluation work clusters in a limited set of clinical fields and that reporting of evaluation design is frequently incomplete [22]. A scoping review of oncology extraction reached a similar conclusion, identifying rapid growth alongside inconsistent definitions of accuracy and limited external validation [23]. Locally deployed open-weight models have been shown to extract structured clinical features from free-text records with high sensitivity and specificity without transmitting text beyond institutional infrastructure [24], and privacy-preserving deployment strategies of this kind are now regarded as a precondition for secondary use of health records with generative models [25]. Occupational rehabilitation is absent from this literature [26]. Machine learning (ML) applied to RTW has focused on outcome prediction from structured variables rather than on extraction from clinical narrative [27], leaving the documentation itself unexamined as a data source.

The absence of empirical research on LLMs in workplace injury assessment represents a significant gap, given the high societal stakes of decisions informed by chart review. Inaccurate or biased data extraction could influence injury classification, treatment recommendations, and compensation outcomes, with disproportionate impacts on marginalized worker populations [28].

This study aims to address the identified knowledge gap through a pilot investigation with the following objectives: (1) assess the accuracy, sensitivity, and specificity of LLMs in extracting clinical-rehabilitative outcomes and sociodemographic data from WSIB medical charts at Trillium Health Partners (THP), Canada; (2) analyze demographic differences in AI-driven classifications of workplace injuries, particularly for marginalized worker populations; (3) examine the ethical and methodological implications of using LLMs in health care decision-making and workplace compensation claims processing; and (4) develop recommendations for integrating AI in clinical-rehabilitative programs to improve quality of care, while ensuring ethical rigor.

This protocol describes the planned methodology for a pilot study that will serve as a proof-of-concept investigation, generating foundational evidence for responsible, reproducible, and equitable AI integration in occupational health clinical workflows.

## Methods

### Study Design

This study will use a retrospective chart review design to evaluate the feasibility and performance of LLM-based clinical data extraction from workplace injury medical records. The study design will follow a proof-of-concept framework, beginning with a pilot sample to establish baseline model performance before proceeding to the full dataset. The study is registered with the Data Sciences Institute at the University of Toronto (grant number DSI-CIDSY4R2P01).

### Setting and Data Source

Data will be obtained from electronic medical charts within the WSIB specialty programs at THP, a large hospital system in the Greater Toronto Area, Ontario, Canada. THP offers seven specialty recovery programs for workplace injuries, funded by the WSIB: Back and Neck, Shoulder and Elbow, Hand and Wrist, Neurology, Foot and Ankle, Hip and Knee, and COVID-19 Assessment Program (CAP). Each program focuses on injury-specific rehabilitation to facilitate rapid recovery and RTW. Previous analyses have demonstrated that participation in four of the seven programs (Back and Neck, Shoulder and Elbow, Hand and Wrist, and Neurology) is effective in reducing depression and anxiety, although these analyses did not address outcomes in the context of RTW [4]. Table 1 provides an overview of the WSIB specialty programs at THP.

For this pilot study, we will begin with 50 retrospective medical charts from the WSIB Back and Neck specialty program, selected because records in this program are typically more concise, thereby reducing the time required for initial chart review and model evaluation. Records will span from January 2018 to December 2024. The broader dataset comprises approximately 1738 records across all WSIB specialty programs and will be used in subsequent phases pending pilot results.

**Table 1:** WSIB^a^ specialty programs at THP^b^.

Program	Target population
Back and Neck	Workers with injuries to the cervical, thoracic, and lumbar spine
Shoulder and Elbow	Workers with injuries to the upper extremities, including the shoulder and elbow
Hand and Wrist	Workers with injuries to the hand or wrist
Neurology	Workers with head injuries, including mild traumatic brain injury and concussion
Foot and Ankle	Workers with injuries to the foot and ankle
Hip and Knee	Workers with injuries to the lower extremities, including the hip and knee
CAP^c^	Workers with WSIB-approved COVID-19 claims post-COVID infection

^a^WSIB: Workplace Safety and Insurance Board.

^b^THP: Trillium Health Partners.

^c^CAP: COVID-19 Assessment Program.

### Variables and Data Extraction

A standardized extraction schema will be developed to capture clinical, rehabilitative, and sociodemographic variables from each medical chart. Table 2 presents the categories of variables targeted for extraction.

**Table 2:** Categories of variables targeted for extraction from WSIB^a^ medical charts.

Category	Variables
Sociodemographic	Age, sex, gender, language, occupation, industry, employment status
Injury characteristics	Injury type, mechanism of injury, body region, date of injury, diagnosis codes
Clinical assessment	Pain severity, functional limitations, range of motion, cognitive status, comorbidities
Treatment and rehabilitation	Treatment modalities, rehabilitation goals, number of sessions, duration of program
RTW^b^ outcomes	RTW status at discharge, time to RTW, work modifications, barriers to RTW
Mental health	Depression and anxiety screening scores (baseline and discharge), psychological referrals

^a^WSIB: Workplace Safety and Insurance Board.

^b^RTW: return to work.

### Chart Structure and Data Representation

Charts in the Back and Neck specialty program combine narrative clinical documentation with a structured discharge outcome record. The narrative component includes WSIB referral documentation, the initial assessment, progress notes recorded across the rehabilitation episode, and the discharge report, and it is the principal source for variables such as occupation and industry, mechanism of injury, functional limitations described in clinical language, and treatment content, where a single variable may be described in more than one section of a chart and in different terms by different clinicians. The structured component is a standardized discharge data template completed for each patient, which records administrative and clinical information in coded fields: program and treatment stream; treatment start and end dates; total number of visits; dates of injury and of surgery, where applicable; primary area of injury; physician diagnoses selected from region-specific categories; and confounding factors affecting length of stay, such as depression or anxiety, posttraumatic stress disorder, or financial and transportation barriers, selected from a defined list.

RTW status is likewise captured in coded form at baseline and at discharge, recorded as working full-time or part-time with regular or modified duties or as one of several defined not-working categories, together with a staged rating of RTW readiness. Standardized outcome instruments are administered at program entry and at discharge and recorded in the template as numeric scores. The “depression and anxiety screening scores” listed in Table 2 are the anxiety and depression subscale scores of the Hospital Anxiety and Depression Scale, captured at baseline and discharge. Pain severity is recorded on the Numeric Pain Rating Scale, condition-specific function on the Oswestry Disability Index and the Neck Disability Index for this program, fear of movement on the Tampa Scale of Kinesiophobia, and health-related quality of life on the EQ-5D-3L, alongside measured functional tests, including timed walk, lifting, carrying, and stair tasks. The variables in Table 2 therefore arrive in mixed representation: some are read directly from coded template fields or instrument scores, others must be extracted from free narrative text, and some, such as RTW barriers, may appear in both and in different terms.

Human annotators and language models will work from the same source documents and the same extraction schema, so any difference in performance reflects a difference in extraction rather than a difference in the information available. For each variable in Table 2, the schema will specify the permitted value set, the document sections in which the variable is expected to appear, and the rule to apply when a chart contains repeated or conflicting entries, for example, by taking the most recent value recorded before discharge. A variable that is absent from a chart will be recorded explicitly as not documented rather than left blank, so failure to extract a value that was recorded can be distinguished from correct recognition that no value exists. The completed schema, including field definitions and coding rules, will be released as supplementary material.

### Eligibility Criteria

Eligible records are those of adults injured at work and referred to WSIB specialty programs with board-approved claims. No maximum age will be imposed, although the sample will primarily consist of individuals younger than 65 years of age, given the nature of employment-based injuries. The study will use preexisting clinical records created in the course of care rather than data collected for research purposes. No participant will be contacted, and no clinical intervention will be introduced.

### AI Model Selection and Architecture

We will evaluate four models in their off-the-shelf (no fine-tuning) form: two general-purpose models (Qwen3-VL-8B and InternVL3.5-8B) and two domain-specific biomedical models (MedGemma-27B and LLaMA-3-Meditron-8B). The general-purpose models are included because their broader pretraining may enable stronger performance on nonclinical or semistructured variables, such as sociodemographic information, where domain-specific knowledge is less critical. In addition, the two biomedical models (MedGemma-27B and LLaMA-3-Meditron-8B) will be further evaluated under a fine-tuning setting using the annotated pilot dataset to assess potential gains in extracting clinically nuanced variables. All models will be prompted with structured queries (eg, “What is the patient’s primary diagnosis?”) to extract predefined variables across sociodemographic, clinical, treatment, and mental health domains. Table 3 summarizes the models, their characteristics, and intended roles in this study.

**Table 3:** LLMs^a^ evaluated in this study.

Model	Parameters	Domain	Approach
Qwen3-VL-8B	~9B	General purpose	Without fine-tuning
InternVL3.5-8B	~9B	General purpose	Without fine-tuning
MedGemma-27B	27B	Medical	With and without fine-tuning
LLaMA-3-Meditron-8B^b^	8B	Medical	With and without fine-tuning

^a^LLM: large language model.

^b^LLaMA: Large Language Model Meta AI.

### Human Annotation and Ground Truth

To establish a reference standard for evaluating model performance, human annotation will be performed on the full pilot sample of 50 medical charts. Three independent annotators with clinical or research expertise in occupational health will review each chart using the standardized extraction schema. Annotators will extract the same set of variables targeted by the AI models. Interannotator agreement will be assessed using Cohen κ to evaluate consistency across annotators and to identify variables that require additional clarification or standardization of extraction criteria [29]. Discrepancies will be resolved through consensus discussion. The consensus annotations will serve as the ground truth against which all model outputs will be compared.

### Data Partitioning

Prompt development, fine-tuning, and final evaluation will be carried out on separate, nonoverlapping sets of charts. Using the same records for all three purposes would allow information from the evaluation data to influence model configuration and would yield performance estimates that are optimistically biased and unlikely to reproduce on unseen records. This failure mode is well documented across applied ML and is a recognized source of irreproducible findings [30], and its avoidance is an explicit requirement of current consensus reporting standards for ML-based science [31].

The 50 charts will be partitioned once, before any model is run, into a development set of 10 (20%) charts, a training set of 25 (50%) charts, and a held-out test set of 15 (30%) charts. Partitioning will be performed at the chart level so that no record contributes to more than one set and will be stratified by sex and by broad age band to reduce the chance that a demographic subgroup is absent from the test set. The allocation will be generated with a fixed random seed, which will be reported so that the partition can be reproduced.

The development set will be the only data used to design and revise extraction prompts and to select few-shot exemplars. The training set will be used exclusively for supervised fine-tuning of the two biomedical models. The held-out test set will not be inspected during prompt development or fine-tuning and will be used once, at the end of the study, to produce the reported performance and fairness estimates for all four models under all three extraction approaches. Where fine-tuning requires a validation signal for early stopping or hyperparameter selection, that signal will be obtained by cross-validation within the training set and never from the test set. Should any test chart be examined for an unanticipated reason, the event and the charts involved will be reported alongside the results.

Human annotation will cover all 50 charts, so consensus ground truth will be available for every partition, and annotators will not be informed of partition membership. Reporting will follow the TRIPOD-LLM statement for studies developing or evaluating LLMs in health care [32], together with the TRIPOD+AI (Transparent Reporting of a Multivariable Prediction Model for individual Prognosis or Diagnosis+AI) statement for prediction model reporting [33].

### Model Performance Evaluation

Model performance will be evaluated by comparing AI-extracted data against the human-annotated ground truth. Performance metrics will be selected based on the type of variable being extracted. For binary variables, we will report sensitivity (true-positive rate), specificity (true-negative rate), the positive predictive value (precision), and the *F*_1_-score. For multiclass classification tasks, macro- and microaveraged *F*_1_-scores and confusion matrices will be used. For continuous variables, including numerical clinical measures, we will report the mean absolute error (MAE) and the root mean squared error (RMSE). For text span and entity extraction tasks, such as diagnosis codes and free-text clinical concepts, we will compute token- and span-level precision, recall, and the *F*_1_-score. Following the approach described by Bannett et al [19], we will assess model explainability by qualitatively examining extraction errors to identify systematic patterns of misclassification. Performance will be compared across the two tiers (general-purpose vs domain-specific models) and the three extraction approaches (zero-shot, few-shot, and fine-tuned). All figures reported as final performance will be computed on the held-out test set defined earlier. Performance on the development or training partitions, where reported, will be labeled as such and will not be presented as an estimate of generalization performance.

The primary model performance outcome is the macroaveraged *F*_1_-score across the categorical variables listed in Table 2, computed on the held-out test set and reported separately for each model and each extraction approach. Macroaveraging is used in preference to microaveraging so that variables with few documented instances contribute equally to the primary outcome rather than being dominated by the most frequently documented categories, which also preserves sensitivity to the subgroup effects examined in the fairness assessment. Secondary outcomes are the per variable *F*_1_-score, the MAE and RMSE for continuous variables, the token- and span-level *F*_1_-score for narrative extraction, and the fairness metrics reported in Table 4.

As a descriptive benchmark for interpreting model performance, we will also compute a human reference ceiling, defined as the macroaveraged *F*_1_-score of each individual annotator against the consensus ground truth, averaged across the three annotators and computed on the same test set and the same variables as the model estimates. This places model and human performance on a common scale and indicates how much of the shortfall from perfect agreement reflects the difficulty of the charts themselves rather than a limitation of the models. It is reported as a context for interpretation and is not a component of the progression criterion.

Progression to the larger study will be considered justified if the best-performing model achieves a macroaveraged *F*_1_-score of at least 0.80 on the held-out test set, without evidence of systematic failure on clinically important variables. This threshold is stated as a pragmatic feasibility criterion for deciding whether to proceed to the larger dataset, not as a validated threshold for acceptable clinical performance or for deployment in compensation decision-making; no such threshold has been established for this task. Evidence of systematic failure will be assessed by inspecting per variable performance for the injury characteristics and RTW outcome variables in Table 2, which carry the most direct consequences for compensation and care, and any variable on which extraction fails consistently will be reported and discussed rather than absorbed into the aggregate.

Continuous-variable performance, narrative extraction performance, and subgroup fairness results will be reported as supporting outcomes rather than as components of the progression criterion. This reflects the precision available from a held-out test set of 15 charts, in which subgroup cells in particular will be small, so that committing in advance to numerical disparity thresholds would imply a precision the pilot cannot support. All primary and supporting estimates will be reported with 95% CIs, and where a CI spans the progression threshold, the result will be reported as indeterminate rather than as meeting or failing it. The fairness results will inform the design and analysis plan of the larger study, in which subgroup sample sizes permit meaningful estimation.

**Table 4:** Fairness metrics for algorithmic bias assessment.

Metric	Definition	Assessment
Demographic parity	The proportion of positive predictions should be equal across demographic groups.	Compare positive prediction rates across subgroups
Equalized odds	True- and false-positive rates should be equal across demographic groups.	Compare true- and false-positive rates across subgroups for each variable

### Fairness and Bias Assessment

A critical component of this study will be the assessment of algorithmic fairness in AI-driven data extraction. We will evaluate whether model performance differs systematically across demographic subgroups defined by age, sex, language, occupation type, and industry. Two primary fairness metrics will be applied, as described in Table 4. Fairness metrics will be computed on the held-out test set against the same consensus ground truth used for accuracy. Because subgroup sample sizes within a 50-chart pilot will be small, subgroup estimates will be reported with exact CIs and read as preliminary signals for the larger study rather than as evidence for or against the presence of bias. Systematic reviews of medical language models report demographic disparities across a wide range of tasks and find mitigation methods to be comparatively immature [34], and closed models have been shown to reproduce racial and gender patterns present in clinical text [35], which supports reporting observed disparities transparently rather than only after a mitigation step has been applied.

In addition, qualitative analysis of extraction errors will be conducted to identify potential sources of harm, including misclassification patterns that may disproportionately affect specific worker populations. This assessment will be informed by clinical expertise and contextualized judgment from team members with experience in occupational health and rehabilitation practice.

### Ethical and Social Analysis

Beyond quantitative model evaluation, this study will examine the ethical and social implications of AI-assisted chart review in occupational health. This component will analyze three data sources generated by the study itself: the catalog of extraction errors produced on the held-out test set, the subgroup performance differences produced by the fairness assessment, and the recorded disagreements between annotators, which identify the variables whose meaning is contested even among trained human reviewers.

Analysis will proceed in two stages. First, each error type observed in the test set will be mapped to the care or compensation decision it could plausibly affect. An omitted RTW restriction bears on workplace accommodation, whereas a misclassified injury mechanism bears on claim adjudication. This mapping will produce a structured risk register recording, for each variable in Table 2, the observed error rate, the direction of error, the decision affected, and the party who would bear the consequence. Second, the research team will hold structured analysis sessions in which occupational therapy, clinical, and data science members will review the register together and assess where automated extraction would shift interpretive authority away from clinicians and injured workers and toward the operators of the extraction system. These sessions will be documented, and points of disagreement within the team will be recorded rather than resolved into a single position.

The outputs of this component will be the completed risk register, a list of variables identified as unsuitable for automated extraction without human verification, and a written account of the conditions under which the research team judges LLM-assisted chart review to be defensible in a compensation context. These outputs are intended to inform governance requirements for the larger study rather than to serve as a general endorsement of the method.

### Data Security and Privacy Architecture

Given that this study involves identifiable personal health information (PHI), a secure hybrid architecture will be implemented to enable graphics processing unit (GPU)–accelerated LLM processing, while ensuring that PHI never leaves hospital custody. Two categories of data are distinguished throughout this protocol: (1) identifiable PHI, comprising the original clinical records, which remains within THP at all times, and (2) deidentified health data, that is, the study dataset produced after deidentification, which is the only data transmitted to cloud compute resources. The architecture will separate data custody from compute execution: all identifiable PHI will be retained within THP’s on-premises secure sandbox environment (MCVHDSMAN90), while Amazon Web Services (AWS) cloud resources will be used exclusively as transient compute layers. Figure 1 presents an overview of this architecture. Key architectural features include the following:

- First, a REST API server (EC2 t3.medium) will operate in a private subnet with no public IP address and will serve as the controlled ingress and egress point for all data transmission.
- Second, GPU-enabled LLM inference nodes (EC2 g5.2xlarge or g6e.2xlarge) will perform model fine-tuning and inference in private subnets accessible only through AWS PrivateLink.
- Third, all data transmitted between THP and AWS will be encrypted using Transport Layer Security (TLS) 1.2 or higher with mutual authentication, routed through a client-to-site virtual private network (VPN).
- Fourth, deidentified health data will be decrypted only in memory during processing on inference nodes and will be immediately re-encrypted before return transmission. No health data will be persisted in AWS storage or logs at any point.
- Fifth, all encryption keys will be generated and managed exclusively within the THP infrastructure.
- Sixth, administrative access to cloud resources will be managed through AWS Systems Manager (SSM) Session Manager, eliminating Secure Shell (SSH) access.
- Seventh, all activity will be logged through AWS Control Tower and forwarded to THP’s security information and event management system (Azure Sentinel) for real-time monitoring.

This architecture will ensure compliance with THP enterprise governance requirements and Canadian privacy legislation, while enabling the computational resources necessary for LLM fine-tuning and inference workloads.

**Figure 1:**
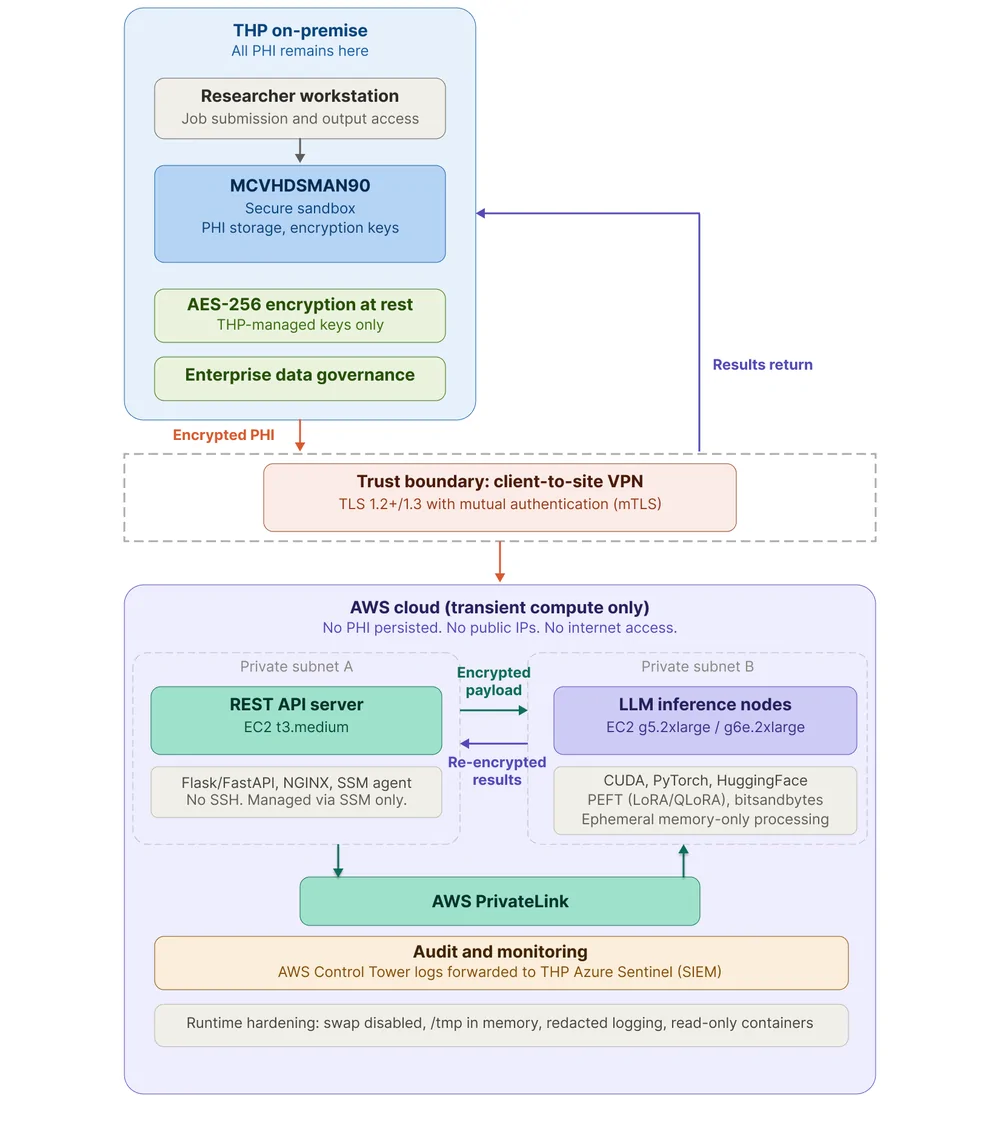
Secure hybrid cloud architecture for the transient processing of WSIB data containing PHI. All PHI remains within the THP on-premises secure sandbox (MCVHDSMAN90). Cloud compute resources in AWS are used exclusively for transient, memory-only processing. Data cross the trust boundary via a client-to-site virtual private network with Transport Layer Security 1.2 or higher and mutual authentication. AWS: Amazon Web Services; PHI: personal health information; SSH: Secure Shell; SSM: Systems Manager; THP: Trillium Health Partners; TLS: Transport Layer Security; VPN: virtual private network; WSIB: Workplace Safety and Insurance Board.

### Ethical Considerations

This manuscript describes a planned study. No chart has been accessed, transferred, or processed, and no data collection will begin before research ethics approval is in place. Approval will be obtained from the research ethics boards of both THP, which holds custody of the records, and the University of Toronto, prior to any data access at either site. The protocol will not proceed to data collection under any other authorization.

Consent provided by a worker to participate in a treatment program is not consent to the research use of that worker’s record, and this protocol does not treat it as such. Both applications will therefore request a waiver of the requirement to seek individual consent for secondary use of identifiable information under Article 5.5A of the Tri-Council Policy Statement [36]. The grounds for the request are that the records were created for clinical and compensation purposes between 2018 and 2024, that many workers completed their programs several years ago and are no longer in contact with the hospital, that recontacting individuals across a 6-year retrospective window is impracticable, that the research could not reasonably be carried out without the waiver, and that the safeguards described next limit residual risk to participants. Whether these grounds are met is a determination for the research ethics boards, and the study will not proceed if the waiver is refused.

Records will be identifiable at the point of access, because sociodemographic and clinical detail must be read from the original documents. Deidentification will therefore be performed inside the THP secure environment before any content enters the processing pipeline described earlier. Direct identifiers will be removed and replaced with study codes, the linking file will be held only within hospital infrastructure and will be accessible only to hospital-based members of the team, and no identifiable content will cross the trust boundary at any stage. References elsewhere in this protocol to deidentified data describe the state of the data after this step rather than at the point of retrieval.

Two distinct retention periods apply, and this distinction matters because the study draws on records maintained for clinical reasons, independent of the research. The deidentified research dataset created for this study will be retained for 5 years, in accordance with THP research data retention policy, after which it will be securely destroyed. The underlying clinical records will remain in the custody of THP and will be retained indefinitely for clinical purposes, as they would be irrespective of this study; the research team will hold no copy of them beyond the research retention period. The file linking study codes to patient identifiers will be destroyed once data collection is complete and annotation has been finalized. Retention periods, storage locations, and destruction procedures will be set out in the ethics applications and followed, as approved.

Data will be accessed, processed, and stored in accordance with the secure hybrid architecture described earlier, so PHI will always remain under hospital custody. Locally deployed open-weight models were selected, in part, for this reason, since they allow inference without transmitting clinical text to an external model provider [24,25]. The research team includes collaborators from THP clinical practice who will contribute expertise in medical chart annotation and interpretation.

## Results

This study was funded in April 2025 by the Data Sciences Institute at the University of Toronto through the Critical Investigation of Data Science Grant program (grant number DSI-CIDSY4R2P01), with a total budget of CAD $10,000 (US $7305) and a duration of 12 months (May 1, 2025, to April 30, 2026). The award supported the formation of the research team and the development of the study plan reported here, which were its stated purposes, and its term has now ended. As of August 12, 2026, neither had a research ethics application been submitted nor had research ethics approval been granted, and no chart data have been accessed, extracted, or analyzed. Applications to the THP and University of Toronto research ethics boards are anticipated in late 2026. Contingent on approval from both boards and on securing funding for the data collection phase, chart retrieval and deidentification, human annotation of all 50 charts, model evaluation on the partitioned dataset, and fairness analysis are anticipated to proceed through 2027, with analysis in late 2027 to early 2028 and results submitted for publication in 2028. These dates are estimates contingent on both conditions being met, and the study will not proceed to data collection until they are.

## Discussion

### Principal Considerations

This protocol describes the first systematic investigation of LLM-based clinical data extraction applied to workplace injury medical charts within a workers’ compensation rehabilitation context. The study will address a critical gap at the intersection of AI in health care, occupational rehabilitation, and algorithmic fairness. By using both general-purpose and domain-specific language models, this study will generate foundational evidence regarding the feasibility, accuracy, and equity implications of automated chart review in occupational health settings.

The model evaluation strategy compares locally deployed open-weight models under both prompting-based and fine-tuning settings. We will assess the off-the-shelf performance of general-purpose models (Qwen3-VL-8B and InternVL3.5-8B) and biomedical models (MedGemma-27B and LLaMA-3-Meditron-8B), as well as the impact of fine-tuning on the biomedical models. This comparison is particularly relevant for clinical settings where the cost and complexity of model fine-tuning must be weighed against the accessibility and flexibility of prompt-based approaches.

### Comparison With Prior Work

Although prior studies have demonstrated the utility of LLMs in clinical data extraction across domains such as pediatric medication monitoring [19], classification of free-text pathology reports [37], and prediction of clinical and operational outcomes from clinical notes [38], no previous work has applied these methods to workplace injury rehabilitation records. The WSIB chart review context introduces domain-specific challenges, including highly variable documentation structures across specialty programs, the integration of clinical and occupational information within single records, and the need to extract variables (eg, RTW barriers and functional capacity assessments) that are not well represented in general biomedical training corpora. This study will provide the first empirical data on LLM performance in this context.

The inclusion of a formal fairness assessment using the demographic parity and equalized odds metrics will also distinguish this study from most existing clinical AI evaluations, which typically focus on aggregate accuracy rather than stratified analyses across demographic groups [20]. Given that workplace injury outcomes are influenced by sociodemographic factors, including age, sex, occupation, and industry, equitable model performance is essential for responsible deployment.

### Limitations

Several limitations should be acknowledged, together with the reasoning behind the design choices that produce them. First, the pilot draws 50 charts from a single specialty program at a single facility, which limits the generalizability of initial findings. Restricting the pilot to the Back and Neck specialty program follows from the annotation burden: consensus ground truth requires three independent annotators to review every chart, and within a 12-month grant of CAD $10,000 (US $7305), the inclusion of a second program would either halve the sample available per program or exhaust the annotation capacity that makes the ground truth credible. Three features of the design limit the cost of this restriction. The extraction schema in Table 2 contains no variable specific to spinal injury and is intended to transfer unchanged to the remaining six programs. Performance will be reported per variable rather than only in aggregate, so variables that depend on program-specific documentation can be distinguished from those that do not. The subsequent phase will draw on the full dataset of approximately 1738 records across all seven programs, and the pilot is structured to produce the schema, the partitioning procedure, and the annotation protocol that this phase requires.

Second, the retrospective design means that documented variables are subject to confounding not captured by the extraction schema. Because the objective is to measure extraction accuracy against what clinicians recorded rather than to estimate causal effects on RTW, this limitation constrains the interpretation of any secondary analysis rather than the primary outcome. Third, a consensus ground truth established by three annotators may not capture the full range of interpretive variability present in clinical chart review. Reporting Cohen κ per variable and retaining the record of preconsensus disagreement will make visible those variables on which human agreement is unstable, and model performance on such variables will be interpreted against a ground truth that is itself uncertain rather than treated as error.

Fourth, the models were not trained on occupational health data, which may limit domain-specific performance. This is the reason the design pairs off-the-shelf evaluation with fine-tuning on the training partition, so the gap attributable to domain mismatch is measured rather than assumed. Fifth, the secure hybrid architecture introduces computational overhead that may affect throughput. This cost is accepted because transmitting clinical text to an external model provider is incompatible with hospital data governance, and processing time will be recorded so that the operational cost of the architecture can be reported alongside accuracy. Sixth, a held-out test set of 15 charts yields small subgroup sample sizes for the fairness analysis. The pilot is designed to detect gross disparities and to establish the measurement procedure rather than to produce precise subgroup estimates, which is a principal reason for proceeding to the larger dataset.

### Conclusion

This protocol outlines a rigorous and ethically informed approach to evaluating LLM-based data extraction in workplace injury rehabilitation. By combining quantitative performance assessment with fairness analysis and critical ethical inquiry, the study aims to establish a foundational methodology for the responsible integration of AI in occupational health. Findings from this pilot will inform the design of a larger-scale study across all WSIB specialty programs at THP, contribute to the development of ethical guidelines for AI-assisted chart review, and support evidence-based policy recommendations for AI deployment in workers’ compensation clinical workflows. The study directly aligns with the Data Sciences Institute’s mission to ensure that data science methodologies are applied in a reproducible, fair, and ethical manner.

## Abbreviations

AI: artificial intelligence
AWS: Amazon Web Services
BioBERT: Bidirectional Encoder Representations from Transformers for Biomedical Text Mining
CAP: COVID-19 Assessment Program
ClinicalBERT: Clinical Bidirectional Encoder Representations from Transformers
EHR: electronic health record
GAI: generative artificial intelligence
GPU: graphics processing unit
LLaMA: Large Language Model Meta AI
LLM: large language model
MAE: mean absolute error
Med-BERT: Medical Bidirectional Encoder Representations from Transformers
ML: machine learning
PHI: personal health information
RMSE: root mean squared error
RTW: return to work
SSH: Secure Shell
SSM: Systems Manager
THP: Trillium Health Partners
TLS: Transport Layer Security
TRIPOD+AI: Transparent Reporting of a Multivariable Prediction Model for individual Prognosis or Diagnosis+AI
VPN: virtual private network
WSIB: Workplace Safety and Insurance Board

## Data Availability

This study will involve retrospective clinical data from Trillium Health Partners (THP) containing personal health information. Data access will be restricted and governed by THP’s data governance policies and the terms of the research ethics approval. Deidentified aggregate results and analysis code will be made available upon publication in a public repository.

## References

[1] Cullen KL, Irvin E, Collie A, Clay F, Gensby U, Jennings PA, Hogg-Johnson S, Kristman V, Laberge M, McKenzie D, Newnam S, Palagyi A, Ruseckaite R, Sheppard DM, Shourie S, Steenstra I, Van Eerd D, Amick BC (2018). Effectiveness of workplace interventions in return-to-work for musculoskeletal, pain-related and mental health conditions: an update of the evidence and messages for practitioners. J Occup Rehabil.

[2] Franche RL, Cullen K, Clarke J, Irvin E, Sinclair S, Frank J (2005). Workplace-based return-to-work interventions: a systematic review of the quantitative literature. J Occup Rehabil.

[3] Nowrouzi-Kia B, Garrido P, Gohar B, Yazdani A, Chattu VK, Bani-Fatemi A, Howe A, Duncan A, Riquelme MP, Abdullah F, Jaswal S, Lo J, Fayyaz Y, Alam B (2023). Evaluating the effectiveness of return-to-work interventions for individuals with work-related mental health conditions: a systematic review and meta-analysis. Healthcare (Basel).

[4] (2024). WSIB specialty programs. Workplace Safety and Insurance Board.

[5] Garies S, Birtwhistle R, Drummond N, Queenan J, Williamson T (2017). Data resource profile: national electronic medical record data from the Canadian Primary Care Sentinel Surveillance Network (CPCSSN). Int J Epidemiol.

[6] Vassar M, Holzmann M (2013). The retrospective chart review: important methodological considerations. J Educ Eval Health Prof.

[7] Thirunavukarasu AJ, Ting DSJ, Elangovan K, Gutierrez L, Tan TF, Ting DSW (2023). Large language models in medicine. Nat Med.

[8] Singhal K, Azizi S, Tu T, Mahdavi SS, Wei J, Chung HW, Scales N, Tanwani A, Cole-Lewis H, Pfohl S, Payne P, Seneviratne M, Gamble P, Kelly C, Babiker A, Schärli N, Chowdhery A, Mansfield P, Demner-Fushman D, Agüera Y Arcas B, Webster D, Corrado GS, Matias Y, Chou K, Gottweis J, Tomasev N, Liu Y, Rajkomar A, Barral J, Semturs C, Karthikesalingam A, Natarajan V (2023). Large language models encode clinical knowledge. Nature.

[9] Lee J, Yoon W, Kim S, Kim D, Kim S, So CH, Kang J (2020). BioBERT: a pre-trained biomedical language representation model for biomedical text mining. Bioinformatics.

[10] Alsentzer E, Murphy JR, Boag W, Weng W-H, Jindi D, Naumann T, McDermott M (2019). Publicly available clinical BERT embeddings. https://aclanthology.org/W19-1909.pdf.

[11] Rasmy L, Xiang Y, Xie Z, Tao C, Zhi D (2021). Med-BERT: pretrained contextualized embeddings on large-scale structured electronic health records for disease prediction. NPJ Digit Med.

[12] Bai S, Cai Y, Chen R, Chen K, Chen X, Cheng Z, Deng L, Ding W, Gao C, Ge C, Ge W, Guo Z, Huang Q, Huang J, Huang F, Hui B, Jiang S, Li Z, Li M Qwen3-VL technical report. arXiv.

[13] Wang W, Gao Z, Gu L, Pu H, Cui L, Wei X, Liu Z, Jing L, Ye S, Shao J, Wang Z, Chen Z, Zhang H, Yang G, Wang H InternVL3.5: advancing open-source multimodal models in versatility, reasoning, and efficiency. arXiv.

[14] Nori H, King N, McKinney SM, Carignan D, Horvitz E Capabilities of GPT-4 on medical challenge problems. arXiv.

[15] Sellergren A, Kazemzadeh S, Jaroensri T, Kiraly A, Traverse M, Kohlberger T, Xu S, Jamil F, Hughes C, Lau C, Chen J, Mahvar F, Yatziv L, Chen T, Sterling B, Baby SA MedGemma technical report. arXiv.

[16] Sallinen A, Solergibert A-J, Zhang M, Boyé G, Dupont-Roc M, Theimer-Lienhard X, Boisson E, Bernath B, Hadhri H, Tran A, Rabbani T, Brokowski T, Rudner TGJ, Hartley M-A, Meditron Medical Doctor Working Group (2025). Llama-3-Meditron: an open-weight suite of medical LLMs based on Llama-3.1. https://openreview.net/pdf?id=ZcD35zKujO.

[17] Chen Z, Cano AH, Romanou A, Bonnet A, Matoba K, Salvi F, Pagliardini M, Fang S, Köpf A, Mohtashami A, Sallinen A, Sakhaeirad A, Swamy V, Krawczuk I, Bayazit D, Marmet A, Montariol S, Hartley M-A, Jaggi M, Bosselut A MEDITRON-70B: scaling medical pretraining for large language models. arXiv.

[18] Touvron H, Lavril T, Izacard G, Martinet X, Lachaux M-A, Lacroix T, Rozière B, Goyal N, Hambro E, Azhar F, Rodriguez A, Joulin A, Grave E, Lample G LLaMA: open and efficient foundation language models. arXiv.

[19] Bannett Y, Gunturkun F, Pillai M, Herrmann JE, Luo I, Huffman LC, Feldman HM (2025). Applying large language models to assess quality of care: monitoring ADHD medication side effects. Pediatrics.

[20] Obermeyer Z, Powers B, Vogeli C, Mullainathan S (2019). Dissecting racial bias in an algorithm used to manage the health of populations. Science.

[21] Rajkomar A, Hardt M, Howell MD, Corrado G, Chin MH (2018). Ensuring fairness in machine learning to advance health equity. Ann Intern Med.

[22] Du X, Zhou Z, Wang Y, Chuang Y-W, Li Y, Yang R, Zhang W, Wang X, Chen X, Guan H, Lian J, Hong P, Bates DW, Zhou L (2026). Testing and evaluation of generative large language models in electronic health record applications: a systematic review. J Am Med Inform Assoc.

[23] Chen D, Alnassar SA, Avison KE, Huang RS, Raman S (2025). Large language model applications for health information extraction in oncology: scoping review. JMIR Cancer.

[24] Wiest IC, Ferber D, Zhu J, van Treeck M, Meyer SK, Juglan R, Carrero ZI, Paech D, Kleesiek J, Ebert MP, Truhn D, Kather JN (2024). Privacy-preserving large language models for structured medical information retrieval. NPJ Digit Med.

[25] Jonnagaddala J, Wong ZSY (2025). Privacy preserving strategies for electronic health records in the era of large language models. NPJ Digit Med.

[26] Neveditsin N, Lingras P, Mago V (2025). Clinical insights: a comprehensive review of language models in medicine. PLOS Digit Health.

[27] Escorpizo R, Theotokatos G, Tucker CA (2024). A scoping review on the use of machine learning in return-to-work studies: strengths and weaknesses. J Occup Rehabil.

[28] Chen IY, Pierson E, Rose S, Joshi S, Ferryman K, Ghassemi M (2021). Ethical machine learning in healthcare. Annu Rev Biomed Data Sci.

[29] McHugh ML (2012). Interrater reliability: the kappa statistic. Biochem Med (Zagreb).

[30] Kapoor S, Narayanan A (2023). Leakage and the reproducibility crisis in machine-learning-based science. Patterns (N Y).

[31] Kapoor S, Cantrell EM, Peng K, Pham TH, Bail CA, Gundersen OE, Hofman JM, Hullman J, Lones MA, Malik MM, Nanayakkara P, Poldrack RA, Raji ID, Roberts M, Salganik MJ, Serra-Garcia M, Stewart BM, Vandewiele G, Narayanan A (2024). REFORMS: consensus-based recommendations for machine-learning-based science. Sci Adv.

[32] Gallifant J, Afshar M, Ameen S, Aphinyanaphongs Y, Chen S, Cacciamani G, Demner-Fushman D, Dligach D, Daneshjou R, Fernandes C, Hansen LH, Landman A, Lehmann L, McCoy LG, Miller T, Moreno A, Munch N, Restrepo D, Savova G, Umeton R, Gichoya JW, Collins GS, Moons KGM, Celi LA, Bitterman DS (2025). The TRIPOD-LLM reporting guideline for studies using large language models. Nat Med.

[33] Collins GS, Moons KGM, Dhiman P, Riley RD, Beam AL, Van Calster B, Ghassemi M, Liu X, Reitsma JB, van Smeden M, Boulesteix A-L, Camaradou JC, Celi LA, Denaxas S, Denniston AK, Glocker B, Golub RM, Harvey H, Heinze G, Hoffman MM, Kengne AP, Lam E, Lee N, Loder EW, Maier-Hein L, Mateen BA, McCradden MD, Oakden-Rayner L, Ordish J, Parnell R, Rose S, Singh K, Wynants L, Logullo P (2024). TRIPOD+AI statement: updated guidance for reporting clinical prediction models that use regression or machine learning methods. BMJ.

[34] Omar M, Sorin V, Agbareia R, Apakama DU, Soroush A, Sakhuja A, Freeman R, Horowitz CR, Richardson LD, Nadkarni GN, Klang E (2025). Evaluating and addressing demographic disparities in medical large language models: a systematic review. Int J Equity Health.

[35] Zack T, Lehman E, Suzgun M, Rodriguez JA, Celi LA, Gichoya J, Jurafsky D, Szolovits P, Bates DW, Abdulnour RE, Butte AJ, Alsentzer E (2024). Assessing the potential of GPT-4 to perpetuate racial and gender biases in health care: a model evaluation study. Lancet Digit Health.

[36] Canadian Institutes of Health Research (CIHR), Natural Sciences and Engineering Research Council of Canada (NSERC), Social Sciences and Humanities Research Council of Canada (SSHRC) (2022). Tri-Council Policy Statement: Ethical Conduct for Research Involving Humans, TCPS 2.

[37] Steinkamp JM, Chambers CM, Lalevic D, Zafar HM, Cook TS (2019). Automated organ-level classification of free-text pathology reports to support a radiology follow-up tracking engine. Radiol Artif Intell.

[38] Jiang LY, Liu XC, Nejatian NP, Nasir-Moin M, Wang D, Abidin A, Eaton K, Riina HA, Laufer I, Punjabi P, Miceli M, Kim NC, Orillac C, Schnurman Z, Livia C, Weiss H, Kurland D, Neifert S, Dastagirzada Y, Kondziolka D, Cheung ATM, Yang G, Cao M, Flores M, Costa AB, Aphinyanaphongs Y, Cho K, Oermann EK (2023). Health system-scale language models are all-purpose prediction engines. Nature.

[39] Suchikova Y, Tsybuliak N, Teixeira da Silva JA, Nazarovets S (2026). GAIDeT (Generative AI Delegation Taxonomy): a taxonomy for humans to delegate tasks to generative artificial intelligence in scientific research and publishing. Account Res.

